# The Effectiveness of Virtual Reality-Based Interventions in Patients with Ataxic Conditions: A Systematic Review with Meta-Analysis

**DOI:** 10.3390/s26072069

**Published:** 2026-03-26

**Authors:** Marina Piñar-Lara, Ana González-Carmona, Esteban Obrero-Gaitán, Irene Cortés-Perez

**Affiliations:** 1 Department of Health Sciences, University of Jaén, Campus Las Lagunillas s/n, 23071 Jaén, Spain; mpl00021@red.ujaen.es (M.P.-L.); icortes@ujaen.es (I.C.-P.); 2 CAIT APROMPSI, C/Clara Campoamor 7, 23470 Cazorla, Spain

**Keywords:** ataxia, virtual reality, virtual reality exposure therapy, disability, postural balance, manual skills, meta-analysis

## Abstract

**Highlights:**

With great interest for clinical practice, this is the first meta-analysis assessing the effectiveness of VRBI as a rehabilitation approach, focused exclusively on patients with ataxia. This meta-analysis reveals that VRBI is effective in improving ataxic symptoms, such as overall disability, balance and manual dexterity.

**Abstract:**

Background: Ataxic symptoms are characterized by causing motor, balance and coordination disorders. Virtual reality-based interventions (VRBIs) including video games and exergames can improve ataxic symptoms. The aim of this systematic review with meta-analysis was to assess the effectiveness of VRBI on severity of ataxia, postural balance, mobility and manual dexterity in patients with ataxia. Methods: According to the PRISMA guidelines, we searched PubMed Medline, SCOPUS, WOS, CINAHL, PEDro and other sources for randomized controlled trials (RCTs) that assessed the effectiveness of VRBI, compared to others, on the severity of ataxia, balance, mobility and manual dexterity in patients with ataxia. The pooled effect was calculated using Cohen’s standardized mean difference (SMD) and a 95% confidence interval (95% CI). Results: With data from seven RCTs, providing data from 171 patients with ataxia, our meta-analysis elucidated that VRBI is effective in reducing the severity of ataxia (SMD = −0.43; 95% CI −0.84 to −0.03; *p* = 0.04) and increasing functional balance (SMD = 0.97; 95% CI 0.16 to 1.78; *p* = 0.02) and manual dexterity (SMD = −0.63; 95% CI −1.16 to −0.11; *p* = 0.018). Conclusions: Our findings suggest that VRBI could be a promising and effective therapeutic approach in reducing ataxia disability and increasing balance and manual dexterity in ataxic patients.

## 1. Introduction

Ataxia is defined as any alteration or anomaly in the interaction of the circuit formed by the cerebellum, basal ganglia, and cerebral cortex [1], causing impairments in muscle tone, motor control, coordination, and postural balance [2]. The initial clinical manifestations are usually gait difficulties, followed by a loss of upper limb and manual dexterity, visual disturbances, and speech disorders, among others [3]. These symptoms impact negatively on the functional capacity and health-related quality of life of these patients [4].

Despite being a heterogeneous group of signs and symptoms, ataxia can be classified into three major groups: acquired, non-hereditary degenerative, and hereditary. The group of acquired ataxias includes those whose pathophysiology involves structural abnormalities (such as stroke, multiple sclerosis or cerebral palsy). The hereditary group is divided into autosomal dominant, exemplified by spinocerebellar ataxias, and autosomal recessive, such as Friedreich’s ataxia and ARCA2, among others [5,6,7]. Recent epidemiological studies show an estimated prevalence of childhood ataxia of 26/100,000. Regarding hereditary ataxia, rates of 2.7/100,000 are reported for dominant variants and 3.3/100,000 for recessive variants [8].

Due to the heterogeneity of ataxia, there is no specific treatment, except in cases of metabolic origin, where pharmacological treatment is effective in halting disease progression [9]. In the remaining cases, lacking disease-modifying drugs, interventions focus on symptom management and the consequences of disease progression [10]. Physiotherapy and neurorehabilitation have demonstrated their potential to significantly reduce motor symptoms without adverse effects [11], as well as to induce improvements in participation in functional activities of daily living [12]. Aerobic and balance training can lead to neuroplastic changes within and outside the cerebellum, which would allow for better compensation of deficits [13], while aerobic training also appears to contribute to combating fatigue-induced symptoms and increasing strength, improving overall stability [14]. For its part, balance training contributes to a better use of afferent input to optimize movement perception [15]. Task-oriented training, as well as performing at least 180 min of weekly exercise, has proven effective in improving balance [16]. The combination of these training types appears to be the treatment of choice: treatment based on multicomponent approaches, working on balance, aerobic capacity, muscle strength, coordination, gait, and activities of daily living training [17]. Furthermore, there is evidence regarding the efficacy of intensive balance and coordination rehabilitation programs in patients with ataxia [18].

Complementary to traditional physiotherapy programs, the design and application of gamified exercises using virtual reality (VR) devices, commercial video games or exergames (video games including physical exercises) is emerging with promising results in patients with neurological disorders [19,20] and could improve ataxic symptoms. VR allows users to recreate a virtual environment using sophisticated hardware and software, which can be recognized as real. According to the level of immersion and presence, VR devices can be non-immersive (NIVR), semi-immersive and immersive (IVR). The application of VR devices, video games or exergames with therapeutic purposes is called virtual reality-based intervention (VRBI). VRBI promotes physical training through different challenges that stimulate motor control, balance, and coordination, which translates into an improvement in function [21] and it has a motivating nature, as VR and exergames promote treatment adherence and generate higher participation rates. The effect of this type of training is also applicable to pediatric populations, where the use of exergames improves signs of progressive ataxia as measured by the Scale for the Assessment and Rating of Ataxia, in addition to producing improvements in quantitative gait analysis [22].

Given the current absence of disease-modifying pharmacological treatments for ataxia, the implementation of rehabilitation strategies plays a crucial role in the symptomatic management of these patients. Specifically, the use of VRBI has been shown to be safe and an effective therapeutic tool to be used in the neurorehabilitation of motor and balance disorders in stroke, multiple sclerosis, cerebral palsy and Parkinson’s Disease, among others [23,24,25,26]. However, a previous systematic review and meta-analysis (SRMA) published in 2021 [27] evaluated the effectiveness of video games, exergames, and mobile technologies for the assessment and rehabilitation of patients with ataxia. That review was limited to six studies, of which only four were experimental and only one was a randomized controlled trial (RCT). Due to this scarcity of high-quality evidence, the meta-analysis could not conclusively determine the effectiveness of VRBI in improving ataxic symptoms. Since 2021, the publication of new RCT studies with greater methodological homogeneity justifies the development of a new SRMA. To overcome the methodological limitations of the prior analysis, we focused only on VRBI on patients with ataxia, with the aim of elucidating the clinical impact of VRBI in these patients with higher precision and statistical power. According to this, a specific literature search was conducted with the aim of retrieving, integrating and synthesizing new RCTs and evidence to assess the effectiveness of VRBI in reducing the severity impact of ataxia symptoms and increasing postural balance, mobility skills and manual dexterity. Additionally, as a secondary objective, the influence of age (pediatric or adult population) in the effectiveness of VRBI was screened, where possible.

## 2. Materials and Methods

### 2.1. Guidelines for Reporting Findings and Protocol Register

The *Cochrane Handbook for Systematic Reviews of Interventions* guidelines and the PRISMA 2020 (Preferred Reporting Items for Systematic Reviews and Meta-Analyses) statement were considered for designing the review and reporting the findings of the meta-analysis [28,29]. To ensure the methodological quality of this SRMA, it was evaluated using the AMSTAR 2 checklist [30]. Finally, the protocol was registered in the PROSPERO database under CRD420251275528.

### 2.2. Literature Search and Databases

To address the research objectives, a systematic and comprehensive search was executed by two authors (M.P.-L. and I.C.-P.), independently, in PubMed Medline, SCOPUS, Web of Science, CINAHL Complete and PEDro (Physiotherapy Evidence Database), capturing all the relevant literature published from inception to 15 December 2025. Additionally, authors screened other sources such as the reference list of previous studies, congress abstracts and proceedings. The main keywords used in the literature search, from Medical Subject Headings (MeSHs), were “virtual reality” and “ataxia”, including related-entry terms. The search was designed using the PICOS framework [31]: (Population) patients with ataxia; (Intervention) virtual reality-based interventions [VRBIs]; (Comparison) conventional physiotherapy or usual/standard care; (Outcomes) clinical outcomes including severity of ataxia, functional balance, mobility skills and manual dexterity; and (Study design) randomized controlled trials [RCTs] or pilot RCTs. Boolean operators (AND/OR) were used to cross the PICOS search conditions (AND) and the terms related with each condition (OR). The search was conducted without using publication date and language restrictions. The search strategies for each database consulted are detailed in Table 1.

### 2.3. Study Selection: Inclusion and Exclusion Criteria

Two authors (M.P.-L. and I.C.-P.), independently, were involved in the study selection process. Study selection was performed in a two-stage process: an initial screening of titles and abstracts followed by a full-text review. A study was included in this SRMA when it met the following inclusion criteria: (1) RCTs or pilot RCTs; (2) studies exclusively comprising patients with ataxia disorders; (3) interventions utilizing VRBI; (4) studies with comparison to conventional physiotherapy or standard care; and (5) availability of quantitative data for meta-analytical synthesis (mean and standard deviation). In contrast, studies including patients with and without ataxia disorders in the same sample were removed. Discrepancies were consulted by a third author (E.O.-G.). The agreement between authors were evaluated as the kappa (κ) coefficient [32]. Agreement was interpreted according to established thresholds: non-existent (κ < 0), non-significant (0 ≤ κ ≤ 0.2), discrete (0.2 < κ ≤ 0.4), moderate (0.4 < κ ≤ 0.6), substantial (0.6 < κ ≤ 0.8), and excellent (0.8 < κ ≤ 1) [33].

### 2.4. Data Extraction

A standardized data extraction protocol was implemented using Microsoft Excel to catalog the following information from each included RCT: (1) overall characteristics (authorship, publication date, country, study design, setting and funding); (2) number of study groups; (3) characteristics of participants (sample size, age, sex, and ataxia type); (4) intervention parameters for the VRBI group (VR modality, video game used, number of sessions, sessions per week and session duration); (5) comparator characteristics (control group protocols); and (6) outcome (measurement tool employed, quantitative data for meta-analysis [mean and standard deviation] and qualitative findings [intra-/intergroup *p*-values] during post-intervention). To ensure accuracy and minimize bias, data extraction was conducted, independently, by two reviewers (M.P.-L. and I.C.-P.). Any inconsistencies were adjudicated through consensus or consultation with a third investigator (E.O.-G.).

### 2.5. Outcomes

The primary clinical outcomes evaluated in this synthesis comprised self-reported severity of ataxia, functional balance, mobility skills and manual dexterity. These variables were systematically analyzed to determine the effectiveness of the VRBI.

### 2.6. Assessment of Risk of Bias and Quality of Evidence

The risk of bias of the included RCTs was rigorously evaluated using the Cochrane Risk of Bias Tool (RoB-1) [28,34]. This scale includes seven items (sequence generation, allocation concealment, blinding of participants, personnel and evaluators, incomplete outcome data, selective reporting and other biases) to assess selection, performance, detection, attrition, reporting and other biases. For each item, risk is scored as low (“+”), high (“−”), and unclear (“?”).

Furthermore, the certainty of the evidence for each meta-analysis was established via the GRADE framework and the Meader (2014) checklist [35,36], assessing five dimensions: overall risk of bias, inconsistency (statistical heterogeneity), imprecision, indirect evidence and risk of publication bias. According to this, the quality of evidence of each finding can be high (when findings were robust and all criteria were met), moderate (when the interpretation or magnitude of the effects could change with the inclusion of additional studies), low (indicating limited confidence in the estimates), or very low (reflecting substantial uncertainty, with four or more criteria not fulfilled). The certainty of evidence was downgraded by one level for each unmet criterion.

These assessments were conducted by two authors (A.G.-C. and M.P.-L.), independently, and disagreements were resolved by a third author (E.O.-G.).

### 2.7. Data Analysis

Meta-analyses (quantitative synthesis) were executed by two independent investigators (E.O.-G. and I.C.-P.) utilizing Comprehensive Meta-Analysis software version 4 (Biostat, Englewood, NJ, USA). To perform the meta-analysis, at least two comparisons per variable were required. The pooled effect sizes were estimated using Cohen’s standardized mean differences (SMDs) and 95% confidence intervals (95% CIs) through a random-effects model, which accounts for expected inter-study clinical and methodological heterogeneity [37,38]. Effect size magnitudes were interpreted according to Kinney et al.’s study for rehabilitation research: null (SMD = 0), small (0.08 ≤ SMD ≤ 0.15 [Cohen’s SMD = 0.2]), medium (0.19 ≤ SMD ≤ 0.36 [Cohen’s SMD = 0.5]), and large (0.41 ≤ SMD ≤ 0.67 [Cohen’s SMD > 0.8]) [39,40]. Findings were visualized via forest plots [41], while potential publication bias was rigorously evaluated through a tripartite approach: visual inspection of funnel plot asymmetry, Egger’s regression test (*p* < 0.1), and the Duval and Tweedie trim-and-fill method [42,43,44]. The trim-and-fill method allowed us to estimate the pooled effect considering a possible risk of publication bias (if the difference between the original and adjusted pooled effect is larger than 10%, downgrading the quality of evidence by one level) [45]. Statistical heterogeneity was screened using the chi-square test (with *p* < 0.1 denoting significance) and Higgins’ I^2^ statistic, where inconsistency was stratified as non-existent (0%), low (10–25%), moderate (25–50%), or high (>50%) [46,47].

Other additional analyses were: (1) sensitivity analysis using the leave-one-out method; (2) meta-regression analysis between pediatric and adult populations; and (3) a qualitative synthesis of the variables of interest through intra- and intergroup *p*-value differences.

## 3. Results

### 3.1. Study Selection

**Figure 1:**
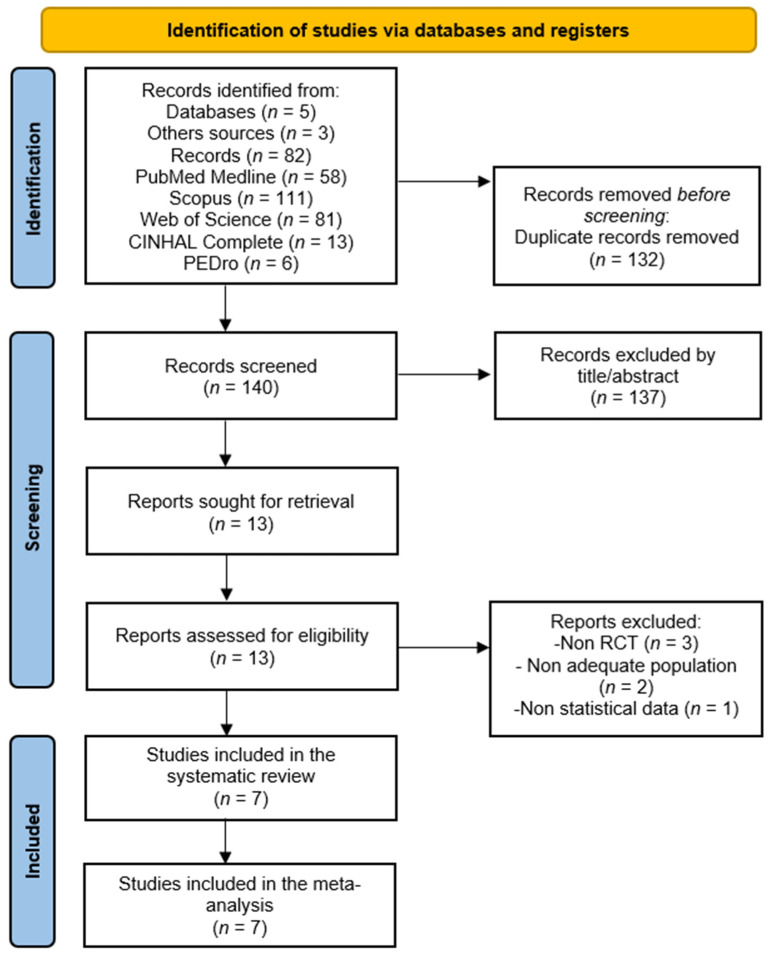
PRISMA 2020 flow diagram for the study selection process (see Supplementary Material).

A total of 269 records were retrieved across five databases (PubMed Medline (*n* = 58), Scopus (*n* = 111), Web of Science (*n* = 81), CINAHL Complete (*n* = 13), PEDro (*n* = 6)), and 3 from other sources. After removing duplicates, 140 records remained for title and abstract screening. Of these, 137 were excluded for not being relevant, and 6 were excluded for not meeting the PICOS inclusion criteria (reasons in Figure 1). Finally, seven RCTs were included in this SRMA [48,49,50,51,52,53,54]. The level of agreement between authors was excellent (k = 0.88).

### 3.2. Characteristics of the Studies Included in This SRMA

The RCTs included were conducted between 2017 and 2025 in countries such as Turkey, Egypt, China, India, Italy and Spain. A total of 171 patients with ataxia (mean age of 35.8 ± 18.8 years old; 55% female) were provided by the RCTs included. The experimental group (receiving VRBI) comprised 87 patients with ataxia (34.5 ± 18.9 years old), while the comparison group (receiving conventional physiotherapy program of exercises or usual care) integrated 84 patients with ataxia (37.1 ± 20 years old). Related to VRBI, participants used exergames in NIVR devices such as Nintendo Wii Balance Board, Kinect sensor, USE-IT system and Niurion Kit. VRBI was applied in protocols ranging from 6 to 12 weeks, 2 to 5 sessions per week and 20 to 60 min per session. All assessments were performed at the end of the intervention (post-immediate). Finally, three RCTs received external funding to perform the research [49,50,53]. More details of the RCTs selected, including qualitative findings of the variables of interest, can be consulted in Table 2.

### 3.3. Evaluation of the Risk of Bias of the Studies Included in This SRMA

**Figure 2:**
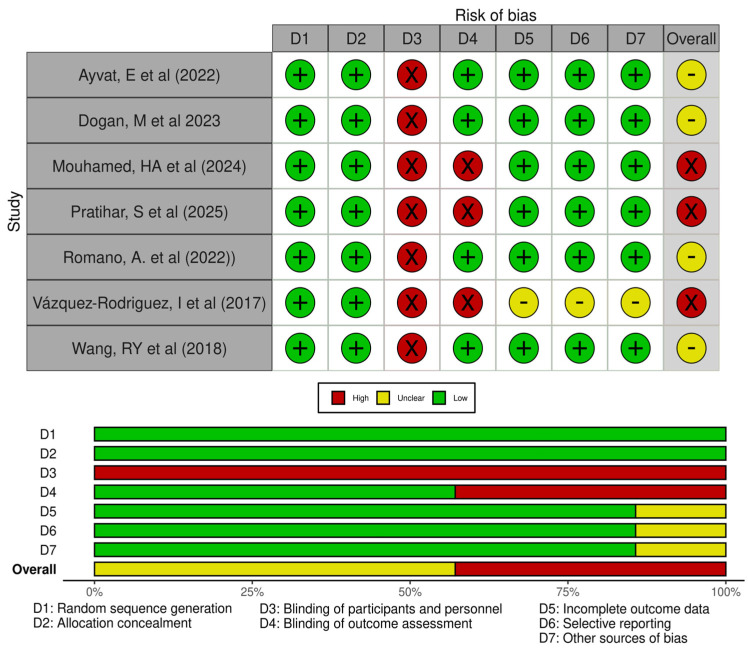
ROB-1 assessment [48,49,50,51,52,53,54].

The overall risk of bias in the RCTs included in this SRMA was moderate–high. Risk of selection bias is not present in any RCT, as items 1–2 were met by all RCTs. Performance bias was present in all RCTs, due to participants and therapists not being blinded. Detection bias, due to non-blinded assessors, was detected in three RCTs (43% of all) [51,52,54]. Additionally, only in the study of Vázquez-Rodríguez and Amezcua-Jaén was the risk of attrition reporting biases unclear. Figure 2 shows the complete RoB-1 assessment.

### 3.4. Quantitative Findings: Meta-Analyses

The main findings of the meta-analyses including GRADE assessment for the quality of evidence are summarized in Table 3.

#### 3.4.1. Severity of Ataxia

**Figure 3:**
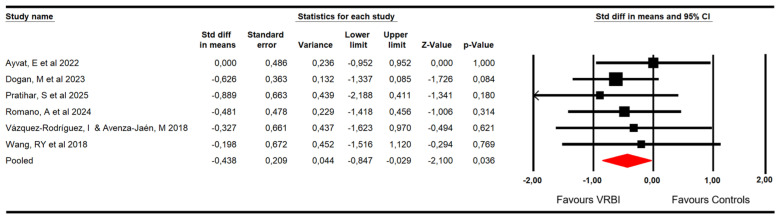
Forest plot of the effectiveness of VRBI on the severity of ataxia [48,49,50,51,53,54].

Six RCTs [48,49,50,51,53,54] with six independent comparisons provided data from 106 patients with ataxia (17.67 per study). In these RCTs, severity of ataxia was measured using the International Co-Operative Ataxia Rating Scale (ICARS) and the Scale for the Assessment and Rating of Ataxia (SARA) assessments. The meta-analysis reported low-quality evidence of a large effect (SMD = −0.43; 95% CI −0.84 to −0.03; *p* = 0.036; I^2^ = 0%; Q = 1.7; df = 5; *p* = 0.88) favoring VRBI (Figure 3). No risk of publication bias was found (Egger *p* = 0.83 and no variation in pooled effect after trim-and-fill calculation). Meta-regression did not show statistically significant differences between pediatric and adult populations (*p* = 0.79).

#### 3.4.2. Functional Balance

**Figure 4:**
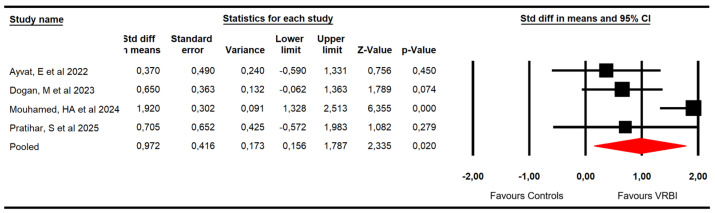
Forest plot of the effectiveness of VRBI on functional balance [48,50,51,52].

**Figure 5:**
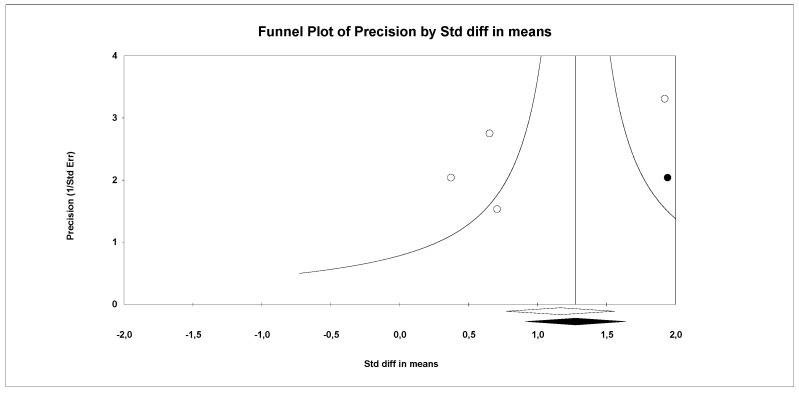
Funnel plot of the effectiveness of VRBI on functional balance’s meta-analysis.

Four RCTs [48,50,51,52] with four independent comparisons reported data from 123 patients with ataxia (30.8 per study). Functional balance was assessed with data from the Berg Balance Scale (BBS), the Pediatric Balance Scale (PBS), the Trunk Impairment Scale (TIS), and the Mini-BESTest. Our findings showed very low-quality evidence of a large effect (SMD = 0.97; 95% CI 0.16 to 1.78; *p* = 0.02; I^2^ = 0%; Q = 2.3; df = 3; *p* = 0.51) favoring VRBI (Figure 4). Risk of publication must be considered in this meta-analysis (Egger *p* = 0.16). Trim-and-fill estimation reported an adjusted pooled effect 19.5% larger (adjusted SMD = 1.16; 95% CI 0.45 to 1.86) than the original, suggesting that publication bias was underestimating the findings (Figure 5). Meta-regression did not show statistically significant differences between pediatric and adult populations (*p* = 0.88).

#### 3.4.3. Mobility Skills/Dynamic Balance

**Figure 6:**
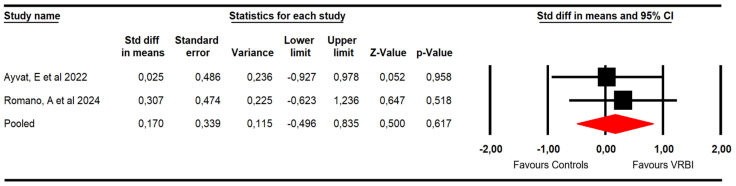
Forest plot of the effectiveness of VRBI on mobility skills/dynamic balance [48,49].

Two RCTs [48,49] with two independent comparisons reported data from 35 patients with ataxia (17.5 per study). Mobility skills were assessed from the Timed Up and Go Test (TUG) and the Timed 25-Foot Walk (T25FW) assessments. This meta-analysis did not find statistically significant differences between groups (SMD = 0.17; 95% CI −0.5 to 0.84; *p* = 0.62; I^2^ = 0%; Q = 0.17; df = 1; *p* = 0.68) (Figure 6).

#### 3.4.4. Manual Dexterity

**Figure 7:**
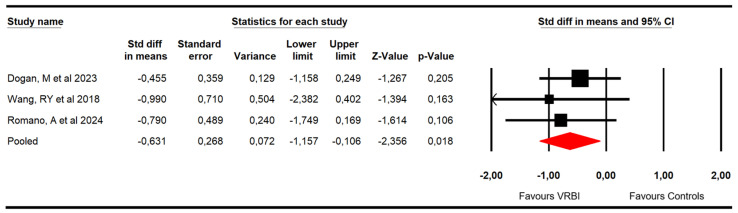
Forest plot of the effectiveness of VRBI on manual dexterity [49,50,53].

**Figure 8:**
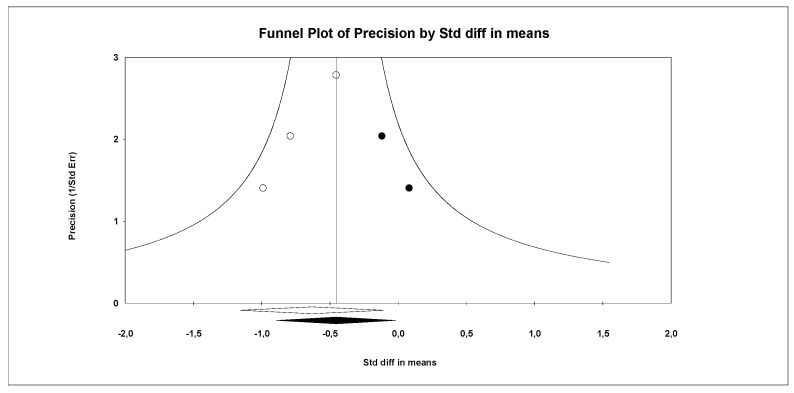
Funnel plot of the effectiveness of VRBI on manual dexterity’s meta-analysis.

Three RCTs [49,50,53] with three independent comparisons provided data from 59 patients with ataxia (19.7 per study). Manual dexterity was evaluated using the Nine-Hole Peg Test (9HPT) and ABILHAND scores. Meta-analysis reported very low-quality evidence of a medium–large effect (SMD = −0.63; 95% CI −1.16 to −0.11; *p* = 0.018; I^2^ = 0%; Q = 0.6; df = 2; *p* = 0.74) favoring VRBI (Figure 7). Risk of publication bias was present (Egger *p* = 0.08). The adjusted pooled effect estimated in trim-and-fill analysis (adjusted SMD = −0.45; 95% CI −0.9 to −0.02) was 40% smaller than the original, suggesting that publication bias was overestimating the findings (Figure 8).

## 4. Discussion

The objective of this SRMA was to evaluate the role of VRBI technologies, including video games and exergames, as an effective therapeutic tool for the rehabilitation of patients with ataxia. While a previous review addressed this topic, it was limited to six studies, of which only two were focused on VR [27]. Additionally, that review provided a meta-analysis including data on two non-controlled and non-randomized studies. Consequently, the generalizability of their findings is limited. Our SRMA addresses these deficiencies by incorporating seven recent RCTs (published between 2018 and 2025), providing data from a total of 171 patients with ataxia [48,49,50,51,52,53,54]. All the included RCTs exclusively evaluated the use of NIVR devices as a therapeutic tool compared to conventional intervention programs. Our SRMA provides a more robust, up-to-date, and comprehensive quantitative synthesis of the treatment effect on the severity of ataxia, balance, mobility and manual dexterity in these patients, in contrast to the limited data available in the previous analysis.

The integration of VRBI using video games or exergames into rehabilitation programs has been tested across various neurological pathologies. Although there is significant variability among the protocols used (number of sessions, intervention duration, and outcome measures), most cases show results like those of conventional therapy [55]. Among its benefits, treatment adherence stands out due to the increased motivation provided by therapy gamification, as well as the possibility of home-based use, owing to the portability and low cost of NIVR systems [56]. The results of our SRMA show that VRBIs are more effective than conventional treatment in reducing the severity of ataxia and improving functional balance and manual dexterity; however, results for mobility skills/dynamic balance are not as positive. Our findings suggest clinical relevance, given the large effect size observed in the variables of severity of ataxia, functional balance, and manual dexterity. However, these results should be interpreted with caution due to the small number of included studies and their limited sample sizes. Recruiting patients with ataxia and achieving sample homogeneity represent a significant challenge when conducting clinical trials, given the variability in pathophysiology and the wide diversity of disease subtypes [57].

The first variable evaluated in this meta-analysis was the effectiveness of VRBI in reducing the severity of ataxia. The meta-analysis showed that VRBIs were highly effective in reducing the impact of the disease compared to conventional therapy in patients with ataxia of all ages. This efficacy remained consistent even when the experimental group received a combination of both therapies [51]. Post-intervention SARA and ICARS scores were significantly lower than baseline scores, suggesting that VRBI contributes to halting the progression of ataxia symptoms and even reducing them. Additionally, our findings did not show statistically significant differences between children and adults with ataxic conditions, reporting that the effect of VGBI could be similar. This suggests that VGBI could be an effective therapy to be included in the rehabilitation of pediatric and adult populations.

Cerebellar damage leads to high variability and poor movement precision; consequently, the primary clinical manifestations in patients with ataxia are uncoordinated movements and deficits in balance control [58]. Specifically, the meta-analysis showed a large effect size for VRBI, resulting from a significant increase in functional balance assessments. The instability challenges provided by VR devices, such as the Nintendo Wii Balance Board or Kinect, activate neuroplasticity mechanisms and sensory compensation circuits that induce improvements in balance. The efficacy of NIVR may lie in the use of devices that require body movements to interact with the virtual world; these movements simulate the exercises performed in conventional therapies to improve balance and coordination [59].

The meta-analysis also showed a large effect size for the improvement of manual dexterity. The repetition of upper limb movements during interaction with devices such as Kinect [48,53] or the Niurion Kit [49] has a positive impact on the patient’s perception of autonomy when performing daily bimanual tasks, while improving precision and speed in small gestures, such as buttoning a shirt. This is objectively reflected through the improved scores on the ABILHAND scale and the decreased times in the 9HPT.

However, the effects on mobility skills/dynamic balance are less conclusive; although there was a reduction in the time required to perform the TUG and T25FW, meta-analysis did not report significant improvement favoring VRBI. The devices used in these RCTs utilize either a camera or body-worn sensors to track body movements. This interaction with the virtual environment may be less challenging than interacting with devices such as a pressure platform (e.g., Nintendo Wii Balance Board). Therefore, improving this specific skill might require more targeted rehabilitation strategies or higher treatment dosages.

The large effect size favoring VRBI in reducing the severity of ataxia and improving functional balance and manual dexterity can be attributed to several biomechanical and neurophysiological mechanisms. On the one hand, the multisensory stimulation provided by VRBI (visual, auditory, exteroceptive, and proprioceptive feedback) can promote neuroplasticity processes related to recovery [60]. Specifically, multisensory stimulation is essential for improving balance, making VRBI an excellent intervention for this purpose. Secondly, VRBI allows patients to perform functional postural movements in gamified environments, which can reinforce existing synapses or establish new synaptic connections, further favoring neuroplasticity [61]. Furthermore, Mao et al. reported that VRBI can activate cerebral areas related to the integration and processing of balance inputs, thereby improving spatial orientation and motor function in patients [62]. The systematic repetition of body movements using VR can induce structural changes in the nervous system through neuroplasticity [63]. The immediate biofeedback provided by these devices allows users to correct errors instantaneously, leading to optimal motor learning [64]. Furthermore, the gamification of physical exercise enhances motivation during rehabilitation, resulting in significantly higher therapeutic adherence [65,66]. These findings are relevant for physiotherapists or rehabilitation practitioners as it provides evidence about the inclusion of VR devices, especially NIVR devices, in conventional rehabilitation programs. Regarding the specific NIVR devices, two studies employed the Kinect sensor and three used the Nintendo Wii console. Both systems share similar operating principles, capturing the user’s body movements through motion sensors to allow for physical interaction with the virtual environment. These NIVR systems are the most widely used in clinical practice and research due to their low cost and user-friendliness. Furthermore, they support various commercial games (such as Wii Sports) that promote patients’ physical rehabilitation, benefiting not only motor function but also balance. Gamification provided by these video games or exergames could favor the adherence of these patients, mainly children, to the rehabilitation program. Additionally, VRBI can be complemented with other technologies devices or technologies that can be useful for the improvement of ataxic symptoms, such as immersive VR software and head-mounted displays, wearable sensors or artificial intelligence platforms. For instance, wearable sensors and artificial intelligence hold significant potential to enhance the diagnosis of motor, gait, and balance disorders in patients with ataxia [67,68,69], as well as in other neurological conditions.

Although findings derived from this meta-analysis are relevant for clinical practice, some limitations can be named. A primary limitation of this SRMA is the relatively small number of included RCTs (*n* = 7) and the resulting limited sample size (*n* = 171), which inherently constrains the statistical power, precision, and generalizability of the findings. However, it should be noted that our search strategy was exhaustive, encompassing all RCTs published to date that specifically assess the effectiveness of VRBI in patients with ataxic conditions. Consequently, the scarcity of studies reflects the current state of the literature rather than a limitation of the search process itself. Despite these constraints, the moderate methodological quality, the acceptable risk of bias, and the low heterogeneity observed across the RCTs suggest that these findings provide a preliminary and valuable foundation for this field of rehabilitation. Secondly, the overall moderate risk of bias and the identification of performance and detection biases may influence the original findings, under- or overestimating them [70,71]. Thirdly, the risk of publication bias in two meta-analyses under- and overestimated the original pooled effect. However, the true effect of VRBI was adjusted using the trim-and-fill method. The heterogeneity between VRBI and controls of the RCTs included is other limitation, but this is usual in physiotherapy studies. To end this section, due to the availability of data, a meta-analysis to assess the effectiveness in the long term cannot been conducted (only post-immediate intervention assessment was conducted). Additionally, the high clinical heterogeneity among the included ataxic populations (multiple sclerosis, cerebral palsy, cerebellar or spinocerebellar ataxia and studies mixing all ataxic conditions) prevented the performance of this subgroup analysis. Finally, although meta-regression indicated no significant age-related differences in ataxia severity (*p* = 0.79) or functional balance (*p* = 0.88), the limited number of comparisons for certain outcomes may have constrained the statistical power to detect subtle variations between pediatric and adult cohorts. Future research should focus on conducting larger, high-quality RCTs with standardized VRBI protocols to improve result precision and include long-term follow-ups to determine the sustained effectiveness of VRBI in patients with ataxia. Additionally, it would be interesting to combine VRBI with other novel technologies such as wearable sensors or artificial intelligence rehabilitation programs, which could minimize the impact of ataxic symptoms in these patients.

## 5. Conclusions

In conclusion, this SRMA suggests that VRBI could be an effective and promising intervention for ataxic patients. Our findings suggest, with low-quality evidence, that VRBI reduces the severity of ataxic symptoms and enhances functional balance and manual dexterity in them. Conversely, no significant improvements favoring VRBI were observed in mobility skills or dynamic balance. Consequently, integrating exergames and video games into neurorehabilitation programs represents a viable, evidence-based strategy to improve motor control and functional autonomy in patients with ataxic conditions. However, due to the novelty of this topic for this population and the small number of studies included, these findings should be interpreted with caution.

## Figures and Tables

**Table 1:** Search strategy conducted in each database consulted for this systematic review.

Database	Search Strategy
PubMed Medline	(ataxia[mh] or cerebellar ataxia[mh] or ataxia[tiab] or cerebellar ataxia[tiab] or myoclonic cerebellar dyssynergia[tiab]) and (virtual reality[mh] or exergaming[mh] or virtual reality exposure therapy[mh] or virtual reality[tiab] or exergam*[tiab] or virtual reality exposure therapy[tiab] or video games[tiab] or videogam*[tiab] or Nintendo[tiab] or head-mounted display*[tiab] or Wii[tiab] or Kinect[tiab])
SCOPUS	title-abs-key (“ataxia” or “cerebellar ataxia” or “myoclonic cerebellar dyssynergia”) AND title-abs-key (“virtual reality” or “exergaming” or “virtual reality exposure therapy” or “exergame” or “exergames” or “wii” or “videogames” or “Kinect”)
WOS	TOPIC (*ataxia* or *cerebellar ataxia* or *myoclonic cerebellar dyssynergia*) AND TOPIC (*virtual reality* or *exergaming* or *virtual reality exposure therapy* or *exergame* or *exergames* or *wii* or *videogames* or *Kinect*)
CINAHL Complete	AB (ataxia or cerebellar ataxia or myoclonic cerebellar dyssynergia) AND AB (virtual reality or exergaming or virtual reality exposure therapy or exergame or exergames or wii or videogames or kinect)
PEDro	Ataxia and virtual reality Ataxia and exergaming

Abbreviations: WOS, Web of Science; PEDro, Physiotherapy Evidence Database.

**Table 2:** Characteristics of the studies included in this systematic review with meta-analysis.

Study	VRBI Group		Control Group		Outcome and Test	Qualitative Findings in Individual Studies	
	Sample Characteristics (Number, Age, and Sex Ratio)	Characteristics of the Intervention Group	Sample Characteristics (Number, Age, and Sex)	Characteristics of the Control Group		Intragroup Differences	Intergroup Differences
Ayvat, E et al. (2022) [48] Turkey Single-blinded RCT Setting: Faculty of Physical Therapy and Rehabilitation, Hacettepe University Funding: NR	8 patients with ataxia (spinocerebellar and multiple sclerosis) Age: 26 (21.5–52) years old Sex: 5 F:3 M	NIVR exercises with the Kinect sensor (exergames). Application: 40 min of exergames + 20 min of conventional balance exercises, 3 days a week for 8 weeks	9 patients with ataxia (spinocerebellar and multiple sclerosis) Age: 31 (26–37) years old Sex: 6 F:3 M	Conventional balance exercises Application: 60 min of exercise; 3 days a week for 8 weeks	Severity of ataxia (ICARS)	Statistically significant improvement in VRBI group (*p* = 0.015) but not in control (*p* = 0.184)	NR
					Functional balance (BBS)	Statistically significant improvement in VRBI group (*p* = 0.018) but not in control (*p* = 0.366)	NR
					Mobility/dynamic balance (TUG)	Statistically significant improvement in VRBI group (*p* = 0.041) but no in control (*p* = 0.173)	NR
Doğan, M et al. 2023 [50] Turkey Single-blinded RCT Setting: Department of Neurological Rehabilitation, Faculty of Physiotherapy and Rehabilitation, Hacettepe University Funding: None	17 patients with ataxia (multiple sclerosis) Age: 36 ± 8.2 years old Sex: 11 F:6 M	Balance and coordination exercises with NIVR using the USE-IT system Application: 60 min, 3 days a week for 8 weeks	15 patients with ataxia (multiple sclerosis) Age: 38.8 ± 5.5 years old Sex: 13 F:2 M	Home-based telerehabilitation exercise program includes balance, strengthening, coordination, and stretching exercises Application: 60 min; 3 days a week for 8 weeks	Severity of ataxia (ICARS)	Statistically significant improvement in VRBI (*p* = 0.01) and control group (*p* = 0.045)	Statistically significant differences favoring VRBI group (*p* = 0.02)
					Functional balance (TIS)	Statistically significant improvement in both groups (*p* = 0.01)	Statistically significant differences favoring VRBI group (*p* = 0.001)
					Manual dexterity (ABILHAND)	Statistically significant improvement in VRBI (*p* = 0.03) and control group (*p* = 0.04)	Non-statistically significant differences between groups (*p* = 0.98)
Mouhamed, HA et al. 2024 [52] Egypt Single-blinded RCT Setting: Outpatient clinic of the Faculty of Physiotherapy, Cairo University Funding: None	32 patients with ataxia (cerebral palsy) Age: 10.7 ± 1.3 years old Sex: NR	Balance exercises with NIVR using Nintendo Wii Balance Board, in addition to a complementary conventional physiotherapy program Application: 30 min of NIVR plus 30 min of conventional physiotherapy, 3 days a week for 12 weeks	32 patients with ataxia (cerebral palsy) Age: 11.2 ± 1.4 years old Sex: NR	Conventional physiotherapy program based on balance, stretching and coordination exercises Application: 60 min of treatment; 3 days a week for 12 weeks	Functional balance (PBS)	Statistically significant improvement in both groups (*p* = 0.001)	Statistically significant differences favoring VRBI group (*p* = 0.001)
Pratihar, S et al. (2025) [51] India Non-blinded RCT Setting: SRM School of Physiotherapy, School of Medicine and Health Sciences, SRM Institute of Science and Technology, Chennai, India Funding: None	5 patients with ataxia (cerebellar ataxia) Age: 48.4 ± 6.3 years old Sex: 1 F:4 M	NIVR exercises with Nintendo Wii Balance Board (Wii Fit Plus) in addition to a conventional physiotherapy program Application: 20 min of exergames + 20 min of conventional physiotherapy, 3 sessions per week for 6 weeks	5 patients with ataxia (cerebellar ataxia) Age: 55.4 ± 11 years old Sex: 1 F:4 M	Conventional physiotherapy program based on balance exercises Application: 40 min, 3 sessions per week for 6 weeks	Ataxia (SARA)	Statistically significant improvement in VRBI (*p* < 0.001) and control groups (*p* = 0.005)	Non-statistically significant differences between groups (*p* > 0.05)
					Functional balance (Mini-BESTest)	Statistically significant improvement in VRBI (*p* < 0.001) and control group (*p* = 0.005)	Non-statistically significant differences between groups (*p* > 0.05)
Romano, A. et al. (2022) [49] Italy Single-blinded RCT Setting: Bambino Gesù Children’s Hospital Funding: Yes—Italian Ministry of Health	9 patients with ataxia (non-genetic, Jouber’s, ARCA2, Friedreich and ARSACS’s ataxias) Age: 11.6 ± 3.2 years old Sex: 6 F:3 M	Upper body exercises with NIVR (Niurion Kit) Application: 60 min per session, 5 days a week for 12 weeks	9 patients with ataxia (non-genetic, Jouber’s, ARCA2, Friedreich and ARSACS’s ataxias) Age: 11.66 ± 4 years old Sex: 5 F:4 M	Conventional physiotherapy program for improving motor skills, balance, and functional exercises Application: 60 min per session, 5 days a week for 12 weeks	Ataxia (SARA)	Statistically significant improvement in control group (*p* = 0.02) but not in VRBI group (*p* = 0.18)	Non-statistically significant differences between groups (*p* = 0.31)
					Mobility/dynamic balance (T25FW)	Non-statistically significant improvement in VRBI (*p* = 0.86) and control group (*p* = 0.31)	Non-statistically significant differences between groups (*p* = 0.31)
					Manual dexterity (9HPT)	Non-statistically significant improvement in VRBI (*p* = 0.17) and control group (*p* = 0.37)	Non-statistically significant differences between groups (*p* = 0.08)
Vázquez-Rodriguez, I and Avenza-Jaén, M (2018) [54] Spain Non-blinded RCT Setting: Health Center in the Community of Madrid Funding: None	11 patients with ataxia (progressive cerebellar ataxia) Age: 54.8 years Sex: NR	NIVR exercises using Nintendo Wii, Wii Fit, and Wii Balance Board in combination with Frenkel exercises Application: 20 min of exergames + 30 min of Frenkel exercises, 2 sessions per week for 8 weeks	10 patients with ataxia (progressive cerebellar ataxia) Age: 54.8 years Sex: NR	Coordination and balance Frenkel exercises Application: 30 min per session, 2 sessions per week for 8 weeks	Ataxia (SARA)	NR	Non-statistically significant differences between groups (*p* = 0.48)
Wang, RY et al. (2018) [53] China Single-blinded RCT Setting: Medical center in Taipei Funding: Yes—National Science Council	5 patients with ataxia (spinocerebellar ataxia type 3) Age: 54 ± 2.3 years old Sex: 3 F:2 M	Balance and coordination exercises using NIVR with Kinect sensor Application: 40 min, 3 sessions per week for 4 weeks	4 patients with ataxia (spinocerebellar ataxia type 3) Age: 57 ± 4.3 years old Sex: 2 F:2 M	Conventional balance and coordination exercises Application: 40 min, 3 sessions per week for 4 weeks	Ataxia (SARA)	Statistically significant improvement in VRBI group (*p* < 0.05) but not in control (*p* > 0.05)	Non-statistically significant differences between groups (*p* > 0.05)
					Manual dexterity (9HPT)	Non-statistically significant improvement in VRBI (*p* > 0.05) and control group (*p* > 0.05)	Non-statistically significant differences between groups (*p* > 0.05)

Abbreviations: VRBI, Virtual Reality-based Intervention; RCT, Randomized Controlled Trial; F, Female; M, Male; NIVR, Non-Immersive Virtual Reality; ICARS, International Co-Operative Ataxia Rating Scale; BBS, Berg Balance Scale; PBS, Pediatric Balance Scale; TUG, Timed Up and Go Test; TIS, Trunk Impairment Scale; SARA, Scale for the Assessment and Rating of Ataxia; T25FW, Timed 25-Foot Walk; 9HPT, Nine-Hole Peg Test; NR, Not Reported.

**Table 3:** Main findings in meta-analyses.

Outcome	Findings Summary											Quality Evidence (GRADE)					
	Effect Size						Heterogeneity		Publication Bias								
	K	N	N_s_	SMD	95% CI	*p*	Q (df)	I^2^ *(p)*	Egger *p*	Trim and Fill		Risk of Bias	Incons	Indir	Impr	Pub Bias	Evidence Strength
										Adj SMD	% Var						
Severity of ataxia	6	106	17.7	−0.43	−0.84 to −0.03	0.04	1.7 (5)	0% (0.88)	0.23	−0.43	0%	Moderate	Low	No	Yes	No	Low
Functional balance	4	123	30.8	0.97	0.16 to 1.78	0.02	2.3 (3)	0% (0.51)	0.16	1.16 (0.45 to 1.86)	19.5%	Moderate	No	No	Yes	Yes	Very low
Mobility skills/dynamic balance	2	35	17.5	0.17	−0.5 to 0.84	0.62	0.17 (1)	0% (0.68)	NP	NP	NP	Moderate	No	No	Yes	Possible	Very low
Manual dexterity	3	59	19.7	−0.63	−1.16 to −0.11	0.02	0.6 (2)	0% (0.74)	0.08	−0.45 (−0.9 to −0.02)	40%	Moderate	No	No	Yes	Yes	Very low

Abbreviations: K, number of studies (comparisons) per meta-analysis; N, number of participants included in each meta-analysis; N_s_, average number of participants per meta-analysis; SMD, Cohen’s standardized mean difference; 95% CI, 95% confidence interval; *p*, *p*-value; Q, Q-test; df, degree of freedom; I^2^, degree of inconsistency of Higgins; Adj, adjusted; % var; percentage of variation; Incons, inconsistency; Indir, indirect evidence; Impr, imprecision; Pub, publication; GRADE, Grades of Recommendation, Assessment, Development and Evaluation; NP, not possible to calculate.

## Data Availability

The raw data supporting the conclusions of this article will be made available by the authors on request.

## Supplementary Materials

The following supporting information can be downloaded at: https://www.mdpi.com/article/10.3390/s26072069/s1, PRISMA check list.

## Abbreviations

The following abbreviations are used in this manuscript:

VRBI	Virtual Reality-based Intervention
NIVR	Non-Immersive Virtual Reality
SMD	Standardized Mean Difference
95% CI	95% Confidence Interval
SRMA	Systematic Review and Meta-Analysis
